# Nr4a1 marks a distinctive ILC2 activation subset in the mouse inflammatory lung

**DOI:** 10.1186/s12915-023-01690-3

**Published:** 2023-10-13

**Authors:** Shasha Xu, Yu Zhang, Xingjie Liu, Huisheng Liu, Xinya Zou, Linlin Zhang, Jing Wang, Zhiwei Zhang, Xiang Xu, Mingxia Li, Kairui Li, Shuyue Shi, Ying Zhang, Zhichao Miao, Jie Zha, Yong Yu

**Affiliations:** 1 Department of Hematology, Tongji Hospital, Frontier Science Center for Stem Cell Research, School of Life Sciences and Technology, Tongji University, 1239 Siping Road, Shanghai, 200092 China; 2 Translational Research Institute of Brain and Brain-Like Intelligence and Department of Anesthesiology, Shanghai Fourth People’s Hospital Affiliated to Tongji University School of Medicine, Shanghai, 200081 China; 3 GMU-GIBH Joint School of Life Sciences, The Guangdong-Hong Kong-Macau Joint Laboratory for Cell Fate Regulation and Diseases, Guangzhou Laboratory, Guangzhou Medical University, Guangzhou, China; 4 Department of Hematology, The First Affiliated Hospital of Xiamen University and Institute of Hematology, School of Medicine, Xiamen University, Xiamen, China; 5 Key Laboratory of Xiamen for Diagnosis and Treatment of Hematological Malignancy, Xiamen, China; 6 Wellcome Trust Sanger Institute, Hinxton, Cambridge, CB10 1HH UK

**Keywords:** ILC2 subset, ILC2 activation, Nr4a1, PD-1, scRNA-seq

## Abstract

**Background:**

Group 2 innate lymphoid cells (ILC2s) are critical sources of type 2 cytokines and represent one of the major tissue-resident lymphoid cells in the mouse lung. However, the molecular mechanisms underlying ILC2 activation under challenges are not fully understood.

**Results:**

Here, using single-cell transcriptomics, genetic reporters, and gene knockouts, we identify four ILC2 subsets, including two non-activation subsets and two activation subsets, in the mouse acute inflammatory lung. Of note, a distinct activation subset, marked by the transcription factor Nr4a1, paradoxically expresses both tissue-resident memory T cell (Trm), and effector/central memory T cell (Tem/Tcm) signature genes, as well as higher scores of proliferation, activation, and wound healing, all driven by its particular regulons. Furthermore, we demonstrate that the Nr4a1^+^ILC2s are restrained from activating by the programmed cell death protein-1 (PD-1), which negatively modulates their activation-related regulons. PD-1 deficiency places the non-activation ILC2s in a state that is prone to activation, resulting in Nr4a1^+^ILC2 differentiation through different activation trajectories. Loss of PD-1 also leads to the expansion of Nr4a1^+^ILC2s by the increase of their proliferation ability.

**Conclusions:**

The findings show that activated ILC2s are a heterogenous population encompassing distinct subsets that have different propensities, and therefore provide an opportunity to explore PD-1's role in modulating the activity of ILC2s for disease prevention and therapy.

**Supplementary Information:**

The online version contains supplementary material available at 10.1186/s12915-023-01690-3.

## Background

Group 2 innate lymphoid cells (ILC2s) are members of a family of innate immune cells comprising five subsets–NK cells, ILC1, ILC2, ILC3, and LTi cells–which form the first line against infections [1] and, especially ILC2s, are abundantly dispersed in barrier tissues including airway, skin, gastrointestinal tract and urogenital tract [2]. Activated ILC2s produce copious amounts of type-2 cytokines (e.g. interleukin-5 (IL-5) and IL-13) which respond to non-specific alarmins such as IL-25, IL-33, and thymic stromal lymphopoietin (TSLP) under infection or insults [3, 4]. ILC2s are generated from hematopoietic progenitors that primarily reside in the bone marrow where committed ILC2 progenitors were differentiated from common lymphoid progenitors (CLPs), common innate lymphoid progenitors (CILPs), common helper innate lymphoid progenitors (CHILPs), and innate lymphoid cell progenitors (ILCPs) in a tightly regulated stepwise approach [1] which are dictated by various transcription factors such as Id2 [5], Tox [6, 7], Nfil3 [8], Tcf7 [9], Ets1 [10], Gata3 [11–13], RORα [14, 15], Gfi1 [16] and Bcl11b [17–19].

Initially, ILC2s were discovered implicating in the early immune responses to parasitic infection [20], but now it is becoming clear that they exert pleiotropic functions in various tissues’ homeostasis and physiology, such as mediating tissue repair through amphiregulin [21], regulating thermogenesis by converting white fat into beige fat [22, 23], sensing neuronal factors NMU [24, 25] and VIP [26], and remodeling intestine epithelium by forming Tuft-ILC2 circuit [27, 28]. Many studies have demonstrated the cross-talk between ILC2s and immune cells or non-immune cells. ILC2s can function as initiators of adaptive immunity [29] or as responders to signals such as cytokines, lipid mediators, and hormones produced by immune cells or structured cells [2].

Dysregulation of ILC2 activity is implicated in a wide variety of type 2-mediated diseases, including allergic rhinitis, atopic dermatitis, and asthma [30, 31]. Recently, several studies reported that the frequency of ILC2s was increased in the peripheral blood [32], sputum [33], and airways [34] of asthmatic patients. Their numbers were positively correlated with the severity of asthma as more ILC2s were detected in the blood and sputum of severe asthma compared to those with mild asthmatics [34]. Moreover, it has been reported that the super-enhancers specific to ILC2s were much more active than that of Th2 cells in the asthmatics, supporting a pathogenic role for ILC2s in patients with allergic asthma [35]. Challenging the mouse with protease allergens such as papain, house dust mites, or Alternaria *alternate* has demonstrated that ILC2s are the key players in the initiation, amplification, and modulation of type 2 immune responses in the airways [36, 37].

It has been reported that mouse lung consists of a functional heterogenous ILC2 population in the hemostatic healthy condition [38] or under the challenges such as influenza virus infection [39], helminth infection [40], and alarmin cytokine stimulation [25]. However, the mechanisms and underlying regulatory networks that drive the segregation and the activation pathways of various ILC2 subsets in the lung are not clear.

PD-1 is a potent immune inhibitory receptor and is expressed on lymphocytes including ILC2s [41].PD-1 knockout in mice leads to development of lupus-like autoimmune diseases [42]. Blockade or inhibition of PD-1/PD-L1 signal pathway in ILC2s results in an increase in ILC2 number, type 2 cytokine production and anti-cancer immunity [43–45].

Here, we revealed that four ILC2 subsets existed in mouse inflammatory lung and a specific activation subset was identified based on the expression of transcription factor Nr4a1. Nr4a1^+^ILC2s displayed many signatures such as memory, migration, and wound healing, all of which were not detected in other ILC2 subsets. We further demonstrated that the Nr4a1^+^ILC2s activation and their differentiation process were negatively regulated by molecule PD-1. Understanding the heterogeneity and activation mechanisms of ILC2s will provide a theoretical principle for the treatment of inflammation-related diseases and help to develop new interventions for therapy.

## Results

### The scRNA-seq analysis identifies four lung ILC2 subsets under acute inflammation

**Fig. 1:**
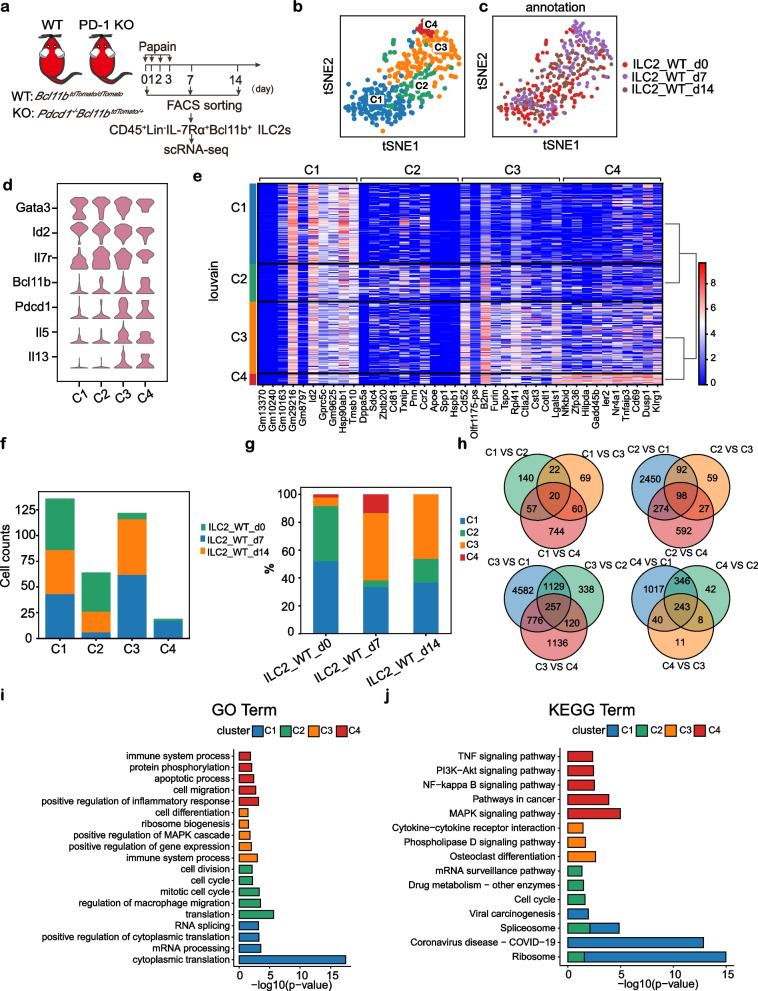
scRNA-seq analysis of mouse lung ILC2s. **a** Experimental design for scRNA-seq of ILC2s sorted from lungs of papain-challenged mice (samples from 2 mice at each timepoint of WT or PD-1 KO mice were mixed). **b** A t-SNE clustering plot showing the grouped clusters of 341 lung-derived ILC2s. Each point represents an individual cell. Cells are colored by clusters. **c** A t-SNE plot showing the time points information about the lung ILC2. Cells are colored by different time points. **d** Stacked violin plots showing the distributions of expression of ILC2-associated genes. The y-axis indicates the log2 (normalized count + 1) expression levels. **e** A heat map shows the expression of selected differentially expressed genes for each ILC2 subset. The Wilcoxon rank sum test is used for analysis. Colors are based on normalized expression levels. **f** The cell composition of each cluster. Colors represent different timepoint cells. **g** Proportions of ILC2 subsets at different time points. Colors represent clusters. **h** Venn diagrams showing the upregulated genes in C1, C2, C3, and C4 respectively by pairwise comparisons (*P* value < 0.05, log2FC > 1). The Wilcoxon rank sum test is used for analysis. **i**-**j** The bar chart shows the GO pathways (**i**) and KEGG (**j**) analysis of DEGs (*P* value < 0.05, log2FC > 1) between four subsets. The colors represent the subsets. The top five terms of each subset are showed

Given the fact that ILC2s are a rare cell population with varying surface makers depending on the mouse genetic background and stimulation, the Bcl11b-tdTomato reporter mice, which label ILC2s in Lin^−^ population of the lung [18, 19], were used for this study. Our previous study shows that PD-1 is expressed on lung ILC2s and its expression is upregulated upon ILC2 activation [41]. We thus purified Lin^−^CD45^+^IL-7Rα^+^ Bcl11b- tdTomato^+^ ILC2s from the lungs of both *Bcl11b*^*tdTomato/tdTomato*^ mice and *Pdcd1*^*−/−*^*;Bcl11b*^*tdTomato/*+^ mice to dissect the heterogeneity of ILC2s and their activation pathways (Fig. 1a and Additional file 1, Fig. S1a). The samples were collected at days 0, 7, and 14 (d0, d7, and d14) after 4 consecutive papain intranasal challenges and were subjected to plate-based smart-seq2 [46] single-cell analysis.

After quality control (Additional file 1, Fig. S1b-d) and the removal of contaminated cells, 703 cells (including 341 WT ILC2s and 362 PD-1 KO ILC2s) were used for this study. We first focus on the analysis of WT cells. Unsupervised clustering grouped them into 4 clusters (clusters 1, 2, 3, and 4, denoted as C1-C4) (Fig. 1b and c) with different gene expression patterns (Fig. 1d and e) by Scanpy [47]. The expressions of ILC2-associated genes such as *Id2, Gata3, Il7r*, and *Bcl11b* validate their ILC2 identity (Fig. 1d). Each cluster consisted of cells from different time points (d0, d7, and d14) at various frequencies (Fig. 1f). In contrast to C1 and C2 clusters that consisted of a majority of d0 and d14 cells, C3, and C4 clusters had the enrichment of d7 cells (Fig. 1f and g), suggesting that C3 and C4 cells were likely activated. Indeed, the C3 and C4 cells had higher expression levels of ILC2 activation-associated genes *Il5*, *Il13*, and *Pdcd1* compared to the C1 and C2 cells (Fig. 1d). Therefore, two non-activation ILC2 subsets and two activation ILC2 subsets were detected in the mouse inflammatory lung.

To characterize C1-C4 subsets, we performed analyses of pairwise differential expression genes (DEGs), Gene Ontology (GO), and Kyoto Encyclopedia of Genes and Genomes (KEGG). We identified 20, 98, 257, and 243 signature genes in C1, C2, C3, and C4 respectively (*P* value < 0.05, log2FC > 1) (Fig. 1h and Additional file 2, Table S1). GO enrichment analysis revealed that several basic biological processes GO terms, including “cytoplasmic translation”, “RNA splicing”, and “mRNA processing”, were overrepresented in C1 signature genes (Fig. 1i), which was consistent with the KEGG analysis that “ribosome” and “spliceosome” were significantly overrepresented (Fig. 1j). The GO terms such as “cell cycle’’, “cell division’’, and “translation” were significantly overrepresented in C2 signature genes (Fig. 1i, Additional file 1, Fig.S1e, and S1f). In line with our previous observations that ILC2 activation-associated genes were highly expressed in C3 and C4 cells, the GO term “immune system process” was overrepresented in their signature genes (Fig. 1i). The GO terms such as “positive regulation of gene expression”, “positive regulation of MAPK cascade”, “cell differentiation” and “ribosome biogenesis” were overrepresented in C3 signature genes, whereas the GO terms including “positive regulation of inflammatory response”, “protein phosphorylation”, “cell migration”, and “apoptotic process” were overrepresented in C4 signature genes, which were consistent with the KEGG pathway analysis (Fig. 1j). Given the absence of C4 cells at d14 coupling with the overrepresentation of GO terms “cell migration’ and “apoptotic process” in their signature genes (Fig. 1f, g and i), we reason that C4 cells might migrate out of the lung or die in it by apoptosis.

Collectively, our scRNA-seq analysis reveals four distinctive ILC2 subsets including two non-activation subsets and two activation subsets in the mouse inflammatory lung.

### Characterization of the transcriptomic signatures of ILC2 activation subsets

**Fig. 2:**
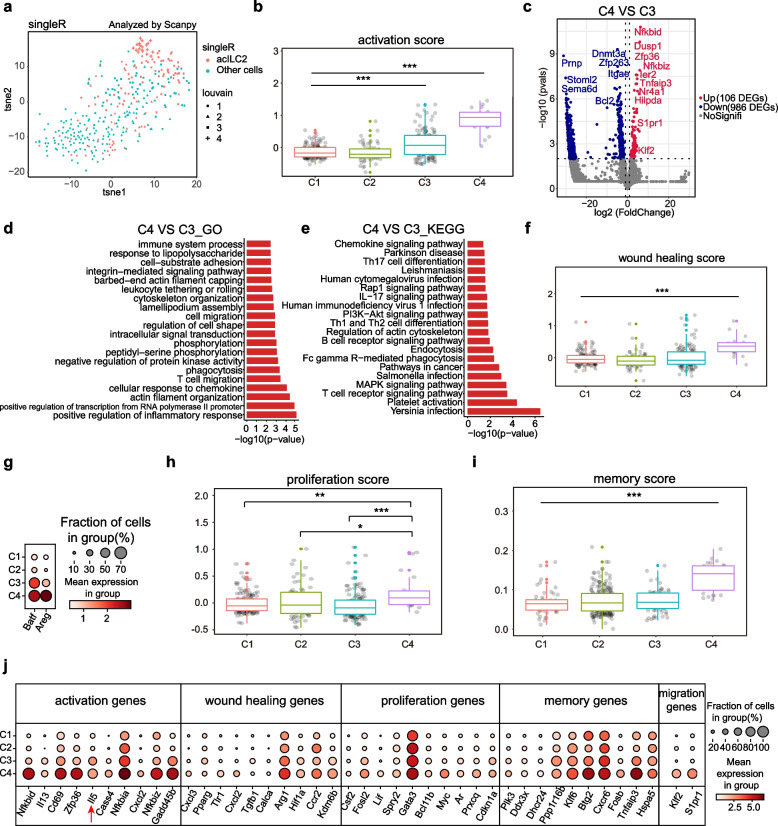
The comparison between C3 and C4 clusters. **a** A t-SNE plot showing the cell state of our 4 ILC2 subsets by of SingleR algorithm annotations based on 3 published mouse lung ILC2s datasets (GSE122762; GSE123994; GSE117567). Red dots indicate activated ILC2s. Blue dots indicate non-activated ILC2s. **b** Box plot shows the distribution of activation scores by cluster. The activation gene sets were sourced from a published study [51]. **c** DEGs in C4 compared with C3 are shown in the volcano plot. *P* < 0.01 and log2FC > 1 were set as the cut-off criterion of significant difference. Red dots indicate the upregulated genes. Blue dots indicate the downregulated genes. **d**-**e** The bar chart shows the GO terms (**d**) and KEGG pathways (**e**) analysis of DEGs (*P* value < 0.05 and log2FC > 1) in C4 compared with C3. **f** Box plot shows the distribution of wound healing scores by cluster. The wound healing score gene sets were sourced from a published report [39]. **g** The dot plot shows distributions of the *Batf* and *Areg* expressions among clusters. **h** Box plot shows the distribution of proliferation scores by cluster. The proliferation gene sets were sourced from a published study [39]. **i** Box plot shows the distribution of memory scores by cluster. The memory gene sets were sourced from a published study [53]. **j** The dot plot shows the distributions of expression of activation, wound healing, proliferation, memory, and migrant genes among clusters. All the signature gene sets can be found in Additional file 3, Table S2

To validate the ILC2 activation subsets, three published mouse lung ILC2s datasets (GSE122762 [48]; GSE123994 [49]; GSE117567 [50]) [38, 51] were integrated by Scanpy [47] and were used as a pre-labeled single-cell reference to annotate our data by SingleR [52]. Almost all C3 and C4 cells fell into the activation subset (Fig. 2a). Therefore, this analysis confirms cells in C3 and C4 were activated.

To interrogate the ILC2 activation subsets, the activation degrees of their cells were assessed based on the ILC2 activation genes signature from the previous report [51]. Both C3 and C4 cells had higher activation scores compared to either C1 or C2 cells (Fig. 2b). Of note, the cells with the highest activation scores were enriched in the C4 (Fig. 2b).

To explore two ILC2 activation subsets in greater detail, we performed the DEGs analysis between these two activation subsets. 986 and 106 genes (*P* value < 0.01, log2FC > 1) were upregulated in C3 and C4 respectively (Fig. 2c). For example, the epigenetic modifier *Dnmt3a*, prion protein *Prnp*, and anti-apoptotic protein *Bcl2* were upregulated in C3 cells (Fig. 2c and Additional file 4, Fig. S2a). The higher expression of *Bcl2* in C3 cells indicated that they have better viability than C4 cells, which is in line with the observations of the existence of C3 cells, but not C4 cells, at d14 (Fig. 1g). Interestingly, the typic immune cell migration genes *Klf2* [54] and sphingosine 1-phosphate receptor 1 (*S1pr1*) [55] were specifically expressed in C4 cells (Fig. 2j).

In contrast to the enrichments of “protein transport”, “nucleocytoplasmic transport”, “translation”, “cytoplasmic translation”, and “ribosomal small subunit export from nucleus” terms in C3 cells (Additional file 4, Fig. S2b and S2c), the C4 cells overrepresented pathways “positive regulation of inflammatory response”, “phosphorylation”, “cell migration”, “TNF signaling pathway”, “MAPK signaling pathway”, and “PI3K-Akt signaling pathway” (Fig. 2d and e).

Previous studies showed that wound-healing genes were enriched in mouse lung ILC2s [21]. We thus scored our lung ILC2s by the expressions of wound healing genes and found that C4 cells scored highest (Fig. 2f). The wound healing genes *Batf* and *Areg* were enriched in C4 compared to other subsets (Fig. 2g) and C4 cells had the highest proliferation score as well (Fig. 2h), implying the C4 cells play a more active role in restoring airway epithelial integrity and tissue homeostasis in acute lung inflammation.

It has been reported that mouse lung ILC2s had the potential to differentiate into memory-like ILC2s [56]. Given the lack of available ILC2 memory signature genes, we scored T cell memory-related genes [53] on our lung ILC2s. We found only C4 cells exhibited T cell memory signature (Fig. 2i). Therefore, C4 cells were likely reported memory ILC2s which respond quicker and stronger upon future challenges with the same or unrelated stimuli [56].

Taken together, we have identified two distinct ILC2 activation subsets. One has better viability that can sustain to resolution phage (detected on both d7 and d14). The other one, which is detected on d7 only, is more activated, more proliferative, and has an enrichment of genes on migration, wound healing, and immune memory (Fig. 2j).

### The transcription factor Nr4a1 marks a distinctive ILC2 activation subset

**Fig. 3:**
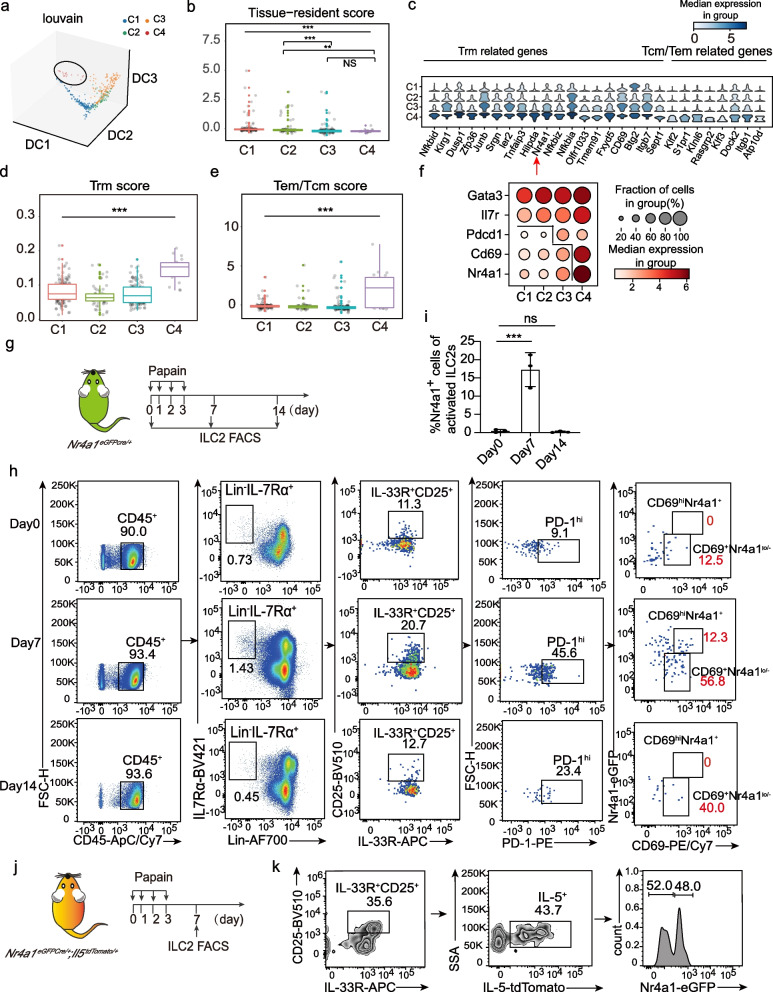
The identification of Nr4a1^+^ ILC2 subset. **a** A 3D plot displaying the distribution of 4 ILC2 clusters by diffusion mapping. DC represents the diffusion component. **b** The box plot shows the distribution of tissue-resident scores by cluster. The gene sets were sourced a previous study [51]. All the signature gene sets can be found in Additional file 3, Table S2. **c** Heatmap showing the representative Trm and Tem/Tcm related DEGs. **d**-**e** Box plot shows the distribution of Trm (**d**) and Tem/Tcm (**e**) scores by cluster. The gene sets were sourced from a previous study [53]. All the signature gene sets can be found in Additional file 3, Table S2. **f** The dot plot shows expressions of the *Gata3*, *Il7r*, *Pdcd1*, *Cd69*, and *Nr4a1* among clusters. **g** Experimental design for C4 cell validation. **h** FACS plots showing the identification of CD45^+^Lin^−^IL-7Rα^+^IL-33R^+^CD25^+^PD-1^hi^CD69^hi^Nr4a1^+^ ILC2 cells in the lung of *Nr4a1*^*eGFPcre/*+^ mice (*n* = 3 mice per group). **i** The percentages of CD69^hi^Nr4a1^+^ cells in activated PD-1^hi^ILC2s at d0, d7, and d14. Data are presented as means ± SEM. Significances between means were analyzed by one-way ANOVA test. **j** Experimental design for Nr4a1^+^ILC2s validation in *Il5*-expressing ILC2s. **k** FACS plots showing the identification of CD45^+^Lin^−^IL-7Rα^+^IL-33R^+^CD25^+^IL-5^+^Nr4a1^+^ ILC2 cells in the lung of *Nr4a1*^*eGFPcre/*+^*Il-5*^*tdtomato/*+^ mice d7 after papain challenge

We projected C1-C4 cells into a 3D graph and found that C4 cells stood out from the others (Fig. 3a). We thus focused on investigating this subset. Although C4 cells had lower ILC2 tissue-resident scores compared to C1 or C2 cells (Fig. 3b), many tissue-resident memory T cells (Trm) associated genes (e.g. *Nfkbid*, *Gadd45b*, *Dusp1*, *Zfp36*, *Junb*, *Tnfaip3*, *Hilpda*, *Nr4a1*, *Cd69*) were significantly enriched in the C4 signature genes (Fig. 3c). Paradoxically, C4 cells also had significantly higher expressions of circulating central memory T cells (Tcm) and effector memory T cells (Tem) associated genes (e.g. *Klf2*, *S1pr1, Klhl6, Rasgrp2, Klf3 Dock2, Itfb1, Apt10d)* (Fig. 3c). We scored both Trm-associated genes and Tcm/Tem-associated genes on activated ILC2s individually. The results showed that C4 cells got both higher Trm and Tcm/Tem scores compared to other cluster cells (Fig. 3d and e), suggesting C4 gains a distinctive feature that is quite different from other subsets.

Compared to other clusters, the nuclear receptor transcription factor *Nr4a1* and surface molecule CD69 were much higher expressed in the C4 (Fig. 3f). We hence utilized the Nr4a1-eGFP reporter [57] to validate the C4 cells by flow cytometry. The Nr4a1-eGFP reporter mice were intranasally challenged with papain and lung tissues were collected at d0, d7, and d14 for analysis (Fig. 3g). In agreement with the transcriptomic data on the dynamic changes of C4 cells in the papain-induced lung inflammation course (Fig. 1g), the CD45^+^Lin^−^IL-7Rα^+^IL-33R^+^CD25^+^PD-1^hi^CD69^hi^Nr4a1^+^ cells were primarily detected on d7 (Fig. 3h and i), suggesting they were C4 cells.

We then generated *Nr4a1*^*eGFPcre/*+^*;Il5*^*tdTomato/*+^ dual reporters mice. Flow cytometry analysis of the lung ILC2s from these mice d7 after papain challenge (Fig. 3j) showed *Il5*-expressing activated ILC2s were demarcated into two populations by the expression of Nr4a1 (Fig. 3k), further confirming there were two activation subsets and the one, marked by the expression of Nr4a1, was C4.

Collectively, the Nr4a1 marks an ILC2 activation subset that has a distinctive dynamic pattern in mouse acute lung inflammation course and exhibits both Trm and Tcm/Tem transcriptome signatures.

### The SCENIC analysis identifies the master regulators for the Nr4a1^+^ILC2 subset

To gain insight into the gene regulatory network driving the unique features of Nr4a1^+^ILC2s from a global perspective, we utilized the single-cell regulatory network inference and clustering (SCENIC) algorithm [58] to identify potential master regulators.

**Fig. 4:**
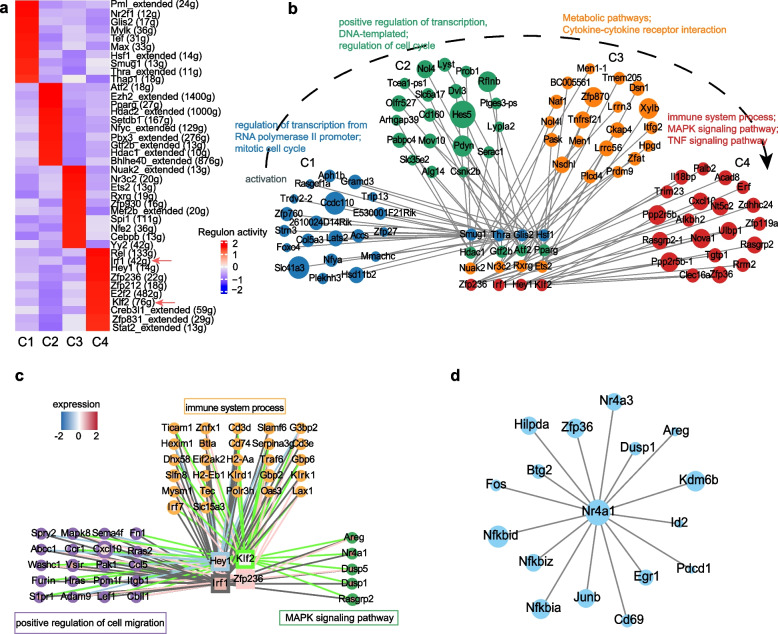
SCENIC analysis identifies the regulators in lung ILC2s. **a** Heatmap shows the main regulons in the C1-C4 subsets respectively. The color key indicates the regulon relative activity. **b**. Graph showing correlation-based gene regulatory network in 4 ILC2 subsets. The nodes in middle represent the top 4 regulators for each indicated cluster. The around nodes represent genes that are directly targeted by the regulators. Different colors represent different clusters. The size of nodes refers to the weight of genes targeted by the indicated transcription factors. **c** Graph showing the top 4 regulons in C4 cells. The 4 nodes in the middle represent transcription factors, and the around nodes represent genes targeted by them. Border paint represents different pathways in which genes are involved. The color inside the node indicates different gene expression levels in C4. The color of the edge indicates different transcription factors. **d** Graph showing correlation-based network of Nr4a1 in ILC2s

We found that Irf1(42g) and Klf2 (76 g) were the top regulons overrepresented in C4 (Fig. 4a and b). One of the direct targets of Klf2 is *S1pr1*, which was reported to have an essential role in ILC2 trafficking between organs [59]. Given the absence of C4 cells in the d14 lung tissue (Fig. 1g), we speculated that the C4 cells might move out of the lung rather than die in it at the resolution phase of inflammation.

Interestingly, the C4 marker gene *Nr4a1*, which was a direct common target of Klf2 and Irf1 regulators (Fig. 4c), directly regulates many C4 signature genes including Trm-associated genes (e.g. *Zfp36*, *Dusp1*, *Nr4a3*, *Hilpda*, *Btg2*, *Fos*, *Nfkbid*, *Nfkbiz*, *Nfkbia*, *Junb*, *Cd69*, *Egr1*), lymphocyte activation marker genes (*Cd69*, *Areg*), and immune inhibitory gene *Pdcd1* (Fig. 4d). Therefore, Nr4a1 is a master regulator of C4 cells.

### PD-1 negatively regulates Nr4a1^+^ILC2s by restraining the cell activation

**Fig. 5:**
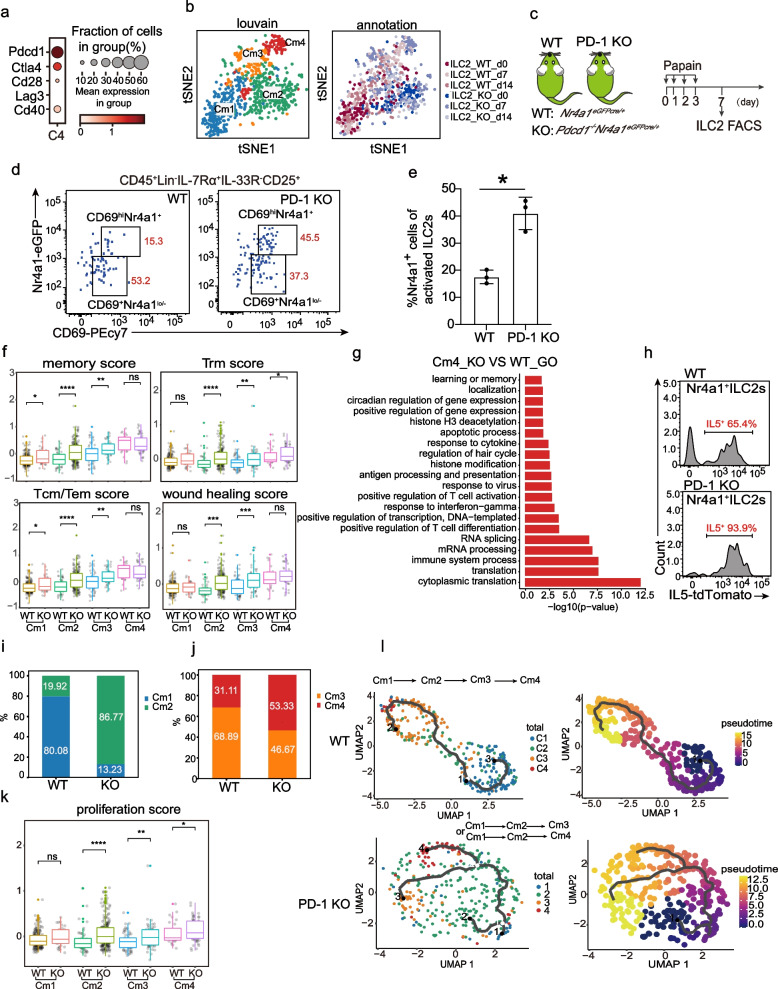
PD-1 is a negative regulator of Nr4a1^+^ILC2s. **a** The dot plot shows the expression of immune checkpoint molecules in Nr4a1^+^ILC2s. **b** Left panel: t-SNE plot showing the clustering of 703 ILC2s (362 KO and 341 WT). Right panel: t-SNE plot showing the time points information about both WT and KO ILC2s. **c** Experimental design for the examination of Nr4a1^+^ILC2s after PD-1 KO. **d** FACS analysis of lung ILC2s of *Nr4a1*^*eGFPcre/*+^ mice and *Pdcd1*^*−/−*^*;Nr4a1*^*eGFPcre/*+^ mice on d7 after papain challenge. **e** The percentages of Nr4a1^+^ILC2s in *Nr4a1*^*eGFPcre/*+^mice (*n* = 3) and *Pdcd1*^*−/−*^;*Nr4a1*^*eGFPcre/*+^ mice (*n* = 3). Data are presented as means ± SEM. The significance between means was analyzed by unpaired t-test. **f** The box plot shows distributions of memory scores, Trm scores, Tcm/Tem scores, and wounding healing scores of clusters from both WT and PD-1 KO ILC2s. **g** The bar chart shows the GO pathways analysis of DEGs (*P* value < 0.05 and log2FC > 1) between PD-1 KO Cm4 and WT Cm4 ILC2s. **h** The percentage of *Il5*-expressing ILC2s in Nr4a1^+^ILC2s from *Pdcd1*^*−/−*^;*Il5*^*tdtomato/*+^ mice or *Il5*.^*tdtomato/*+^mice. **i** Proportions of Cm1 and Cm2 cells in WT non-activation cells or in PD-1 KO non-activation cells. **j** Proportions of Cm3 and Cm4 cells in WT activation cells or in PD-1 KO activation cells. **k** The box plot shows the distribution of proliferation scores of clusters in WT and PD-1 KO ILC2s. **l** The top 2 panels show the WT ILC2 activation trajectory, colored by cluster (left) and inferred by pseudotime (right). The bottom 2 panels show the PD-1 KO ILC2 activation trajectory. The lines correspond to the principal graph learned by Monocle 3

As mentioned above, *Pdcd1* is a direct target of Nr4a1 and is the only immune inhibitory gene examined that is highly expressed in the Nr4a1^+^ILC2 subset (Fig. 5a), we then study the role of PD-1 in Nr4a1^+^ILC2s under acute lung inflammation by using single PD-1 KO ILC2s transcriptome data (Fig. 1a). After the integration of the above 341 WT cells, 703 cells in total were used for further analysis. Similarly, 4 clusters (denoted as Cm1, Cm2, Cm3, and Cm4) were observed in the unsupervised clustering assay (Fig. 5b). We annotated the cells with previous cluster information (C1-C4) on each cell from Cm1-Cm4 clusters and found that almost all WT cells in the Cm1-Cm4 clusters were the same ones from the C1-C4 clusters correspondingly (Additional file 5, Fig. S3a). Thus, Cm3 and Cm4 were two activation subsets with high expressions of activation-associated genes such as *Il5*, *Il13*, and *Areg* (Additional file 5, Fig. S3b), whereas Cm1 and Cm2 were two non-activation subsets. As expected, the C4 marker gene *Nr4a1* was enriched in the Cm4 cluster (Additional file 5, Fig. S3b). The PD-1 KO Cm4 cells were validated in a flow cytometry analysis of the *Pdcd1*^*−/−*^*; Nr4a1*^*eGFPcre/*+^ mice administrated with papain intranasally (Fig. 5c and d). Compared to WT Cm4 cells in *Nr4a1*^*eGFPcre/*+^ mice, more PD-1 KO Cm4 cells were detected in *Pdcd1*^*−/−*^*; Nr4a1*^*eGFPcre/*+^ mice (Fig. 5e).

We then assessed the effects of PD-1 deletion on Nr4a1^+^ILC2s. Gene expression comparison showed PD-1 KO Nr4a1^+^ILC2s had similar scores of memory, Trm, Tem/Tcm, and wound healing relative to control WT Nr4a1^+^ILC2s (Fig. 5f), demonstrating the major signatures of those cells were not changed through PD-1 KO.

Nevertheless, PD-1 KO Nr4a1^+^ILC2s had higher enrichments of the immune system process of GO and KEGG categories (Fig. 5g and Additional file 5, Fig. S3c) and the frequency of *Il5*-expressing cells was increased in those PD-1 KO cells (Fig. 5h). Moreover, gene set enrichment analysis (GSEA) showed that PD-1 deficiency led to a metabolic shift towards glucolipid metabolism in Nr4a1^+^ILC2s (Additional file 5, Fig. S3d), which is consisted with previous reports showing that deletion of PD-1 switched activated ILC2 metabolism to glycolysis [43].

To determine the effects of PD-1 deletion on the cell composition of the clusters, we first calculated the cells in non-activation subsets of WT and PD-1 KO ILC2s. We found that non-activation WT ILC2s comprised 80% of Cm1 cells and 20% of Cm2 cells, whereas non-activation PD-1 KO ILC2s consisted of 13% of Cm1 cells and 87% of Cm2 cells (Fig. 5i). The transcriptome difference existed between WT and PD-1 KO non-activation ILC2s. GO and KEGG analyses revealed PD-1 KO non-activation ILC2s had overrepresentations of immune system process pathways such as “positive regulation of inflammatory response”, “TNF signaling pathway”, “NF kappa B signaling pathway’, and “Oxidative phosphorylation” (Additional file 5, Fig. S3e).

We next calculated the cells in activation subsets of WT and PD-1 KO ILC2s. We found that WT-activated ILC2s comprised 69% of Cm3 cells and 31% of Cm4 cells, whereas PD-1 KO-activated ILC2s consisted of 47% of Cm3 cells and 53% of Cm4 cells (Fig. 5j). The increase in the frequency of PD-1 KO Cm4 cells is compatible with their higher proliferation score relative to the control WT Cm4 cells (Fig. 5k).

Besides the increased proliferating capacity, the increase of PD-1 KO Cm4 cells’ numbers might be also caused by the enhanced Cm4 cell generation from non-activation cells. We then thought to determine whether PD-1 KO promotes Cm4 cell differentiation. We constructed the activation trajectories of ILC2s by monocle pseudotime analysis [60]. The results showed that activation of WT ILC2 followed a Cm1-Cm2-Cm3-Cm4 order, whereas activation of PD-1 KO ILC2 followed Cm1-Cm2-Cm3 or Cm1-Cm2-Cm4 order (Fig. 5l). In the absence of PD-1, Cm2 cells can directly differentiate into Cm4 cells, which was not detected in WT ILC2s. We thus compared the gene expressions between WT and PD-1 KO Cm2 cells and found that PD-1 KO Cm2 cells had significant enrichments of immune response pathways (Additional file 5, Fig. S3f), demonstrating that PD-1 deficiency places the Cm2 cells in a state prone to activation.

In addition, we observed that fewer activated ILC2 cells were detected at d14 after the challenge in *Pdcd1*^*−/−*^ mice (Additional file 5, Fig. S3g), suggesting that PD-1 is involved in regulating the duration of ILC2 activation subsets under acute inflammation.

Taken together, we found that PD-1 is a negative regulator of the Nr4a1^+^ILC2 subset, where PD-1 modulates the activation, differentiation trajectory, duration, and metabolism.

### PD-1 deficiency leads to an upregulation of activation-related regulons in Nr4a1^+^ILC2s

**Fig. 6:**
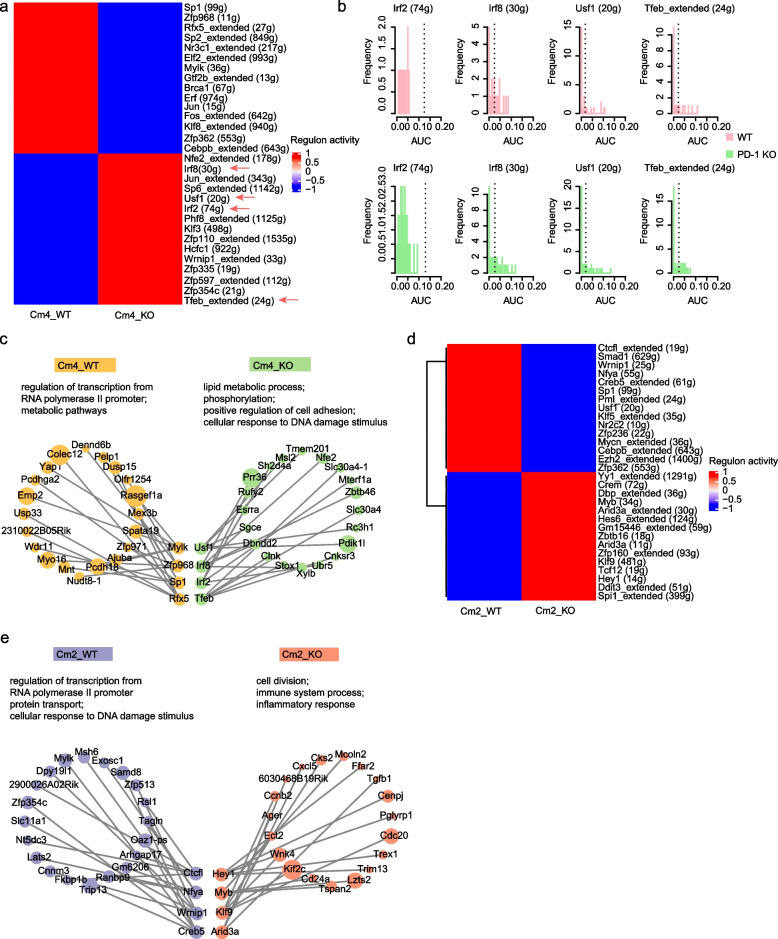
Identification of regulons in ILC2s after PD-1 KO. **a** Heatmap shows the main regulons in Cm4 cells before and after PD-1 KO. The color key indicates the regulon relative activity. **b** Histograms show the comparison of activities of the Irf2 (74 g), Irf8 (30 g), Usf1 (20 g), and Tfeb_extended (24 g) regulons between PD-1 KO and WT Cm4 cells. Plots show frequency (y-axis) versus area under the curve (AUC; x-axis) for each regulon within the indicated subset. The vertical broken line indicates the average AUC score. **c** The graph shows the top 4 regulons in PD-1 KO or WT Cm4 cells. **d** Heatmap shows the main regulons in Cm2 cells before and after PD-1 KO. The color key indicates the regulon relative activity. **e** The Graph shows the top 4 regulons in PD-1 KO or WT Cm2 cells

To investigate the gene regulatory network driving the activation of Nr4a1^+^ILC2s after PD-1 deletion, we performed SCENIC analysis. The overrepresentation of inflammation response associated regulons such as Irf2 (74 g) and Irf8 (30 g) in PD-1 KO Cm4 cells (Fig. 6a, b and c) were in agreement with the above results that PD-1 restrained the Cm4 activation. The enrichments of metabolic pathways related regulons including Tfeb (24 g) [61] and Usf1 (20 g) [62] in PD-1 KO Cm4 cells were compatible with PD-1 regulating the metabolism in Cm4 cells.

Since the deletion of PD-1 had a profound effect on the non-activation Cm2 subset, which resulted in an accelerated activation of the Cm4 cells, we assessed the regulons in the PD-1 KO Cm2 cells. In consistence with the above data (Fig. 5l and Additional file 5, Fig. S3f), the SCENIC analysis identified many regulons that were enriched for the proliferation and inflammatory response in PD-1 KO Cm2 cells (Fig. 6d and e). Altogether, these data show that PD-1 negatively regulates the activation-related regulons in Nr4a1^+^ILC2s.

## Discussion

ILC2s are the major tissue-resident lymphoid cells in the lung mucosa and play essential roles in its hemostasis and inflammatory diseases. By using scRNA-seq combined with the mouse genetics approaches, we identified four lung ILC2 subsets including two activation subsets and two inactivation subsets, which display distinct transcriptomic signatures and have different dynamic patterns during acute papain intranasal challenges. We further demonstrated that PD-1 actively regulate all those subsets as the deletion of PD-1 profoundly affects both the activation subsets and the non-activation subsets in terms of changing their transcriptome and regulons, which lead to the changes in cell activation state and differentiation trajectories.

An activated cluster, marked by Nr4a1 and only detected on day 7 after the papain challenge, clearly stands out from the others and is featured by several unique signatures. One is that they specifically express high levels of migration associated-genes (e.g., *Klf2*, *S1pr1*). ILC2s were initially reported as tissue-resident cells in parabiosis experiments, where the mice were surgically joined to share their bloodstreams and the cell's migratory capacity was examined [63, 64]. However, recent studies in animal models have shown that ILC2s can circulate between organs upon activation by *Nippostrongylus brasiliensis* infection, protease allergen papain challenge, or systemic IL-33 administration [65]. Particularly, one study showed that ILC2s migrate from the lung to the liver after intranasal IL-33 treatment and those lung-derived liver ILC2s produce more IL-5, IL-13, and IL-6 compared to resident lung ILC2s [66]. In line with this data, the Nr4a1^+^ILC2 subset, identified in this study, has the highest activation levels compared to other subsets in the lung upon papain intranasal challenge. Considering the high expression of migration-associated genes in Nr4a1^+^ILC2s, it is very likely that the Nr4a1^+^ILC2s are the cells that migrate into the livers.

The memory T cells signature of Nr4a1^+^ILC2s is in keeping with the earlier findings that allergen-experienced ILC2s acquire memory-like properties in the lung and the draining mediastinal lymph node (mLN) [56]. Given the dynamic of the Nr4a1^+^ILC2 subset in the inflammation course detecting on day 7 and disappearing on day 14 in the lung, we reason that stimulation-induced Nr4a1^+^ILC2s emigrate from the lung in the early phase and migrate into other organs such as mLN [56] and liver [66], where they remain the high expression of IL-5 and IL-13 for a much longer time and regulate the immunity of the organ as demonstrated in the previous study [66]. Moreover, the Nr4a1^+^ILC2s have paradoxical expressions of Trm and Tem/Tcm signature genes, indicating that Nr4a1^+^ILC2s exhibit the residency transcriptomic signature imprinted by the lung but gain the migratory transcriptomic signature upon activation, and further confirming both residual residence nature and circulation nature of these cells.

In addition, the wound healing signature of Nr4a1^+^ILC2s is largely in agreement with their functions in modulating tissue integrity and homeostasis as reported [39]. Given both the residency and migratory natures of the Nr4a1^+^ILC2s, we speculate that these cells play more active roles in various tissues’ homeostasis under systemic infection or insults.

We found that many genes associated with the signatures mentioned above are the direct targets of Nr4a1, which is the marker gene of this activated subset. Nr4a1 is an orphan nuclear receptor that comprises a central two-zinc DNA-binding domain, an N-terminal transactivation domain, and a C-terminal ligand-binding domain, and works as a transcription factor in a ligand-independent manner [67, 68]. Nr4a1 is considered as an immediate-early gene, whose expression is rapidly and transiently induced by a variety of extracellular signals. Indeed, the expression of Nr4a1 in ILC2 was induced by IL-33 administration in vivo [69]. Two recent studies demonstrated that Nr4a1 plays an essential role in Trm cell generation [70, 71]. Given the similarity of gene expression between Nr4a1^+^ILC2s and Trm cells, we reason that Nr4a1 is an important regulator for this unique ILC2 activation subset. The function of Nr4a1 and its orchestrating mechanisms in ILC2 activation warrant further investigation.

Our previous study firstly reported that PD-1 is expressed on ILC precursors and mature ILC2s, where PD-1 expression is distributed from 20 to 40% in various mucosa tissues, and PD-1 expression is substantially increased in the lung ILC2s upon influenza virus infection [41]. The following studies demonstrated that PD-1 is a negative regulator of ILC2 function in cytokine production [72], in *Nippostrongylus brasiliensis* expulsion [45], and anti-tumor immunity [44], and PD-1 also acts as a metabolic checkpoint in ILC2s [43]. In agreement with those data, we found that PD-1 negatively regulates Nr4a1^+^ILC2s by modulating their activation, proliferation, differentiation trajectory, and metabolism. Of note, besides the negative roles of PD-1 in the Nr4a1^+^ILC2 subset itself, PD-1 also functions in its ancestors that are non-activation subsets. Loss of PD-1 sets the non-activation ILC2s in a state prone to activation and leads to a change in activation trajectory that the Nr4a1^+^ILC2s directly differentiated from a non-activation subset without passing another activation subset which has a lower activation score. Moreover, we observed that, in the absence of PD-1, activated ILC2 contraction occurs earlier as much fewer activated PD-1 KO ILC2s were detected at day 14 compared to the activated WT ILC2s after the papain challenge. Thus PD-1 is a negative regulator of ILC2 activation but is a positive regulator of activated ILC2 duration.

In summary, our combined scRNA-seq and genetics mouse analyses of lung ILC2s under acute inflammation enabled the identification of distinct ILC2 subsets in the mouse lung, delineation of lung ILC2 activation trajectories, and characterization of a distinct activation subset marked by Nr4a1, as well as demonstrating the critical role of PD-1 in regulating these subsets. Our study highlights the heterogeneity of lung ILC2s, and the capacity of specific ILC2 subsets to migrate between tissues and organs upon activation. Understanding the molecular mechanisms underlying ILC2 migration will provide a perspective for the development of therapies specifically targeting the migratory population of ILC2s in type 2 immune-mediated diseases.

## Conclusions

The findings show that activated ILC2s are a heterogenous population encompassing distinct subsets that have different propensities, and therefore provide an opportunity to explore PD-1's role in modulating the activity of ILC2s for disease prevention and therapy.

## Methods

### Mice

The *Bcl11b*^*tdTomato/tdTomato*^ knock-in reporter mice were previously reported [19]. *Pdcd1*^*−/−*^ (028276) mice were purchased from Jackson Laboratory. *Pdcd1*^*−/−*^*;Bcl11b*^*tdTomato/*+^ mice were generated by crossing *Pdcd1*^*−/−*^ mice to *Bcl11b*^*tdTomato/tdTomato*^ mice. *Bcl11b*^*tdTomato/tdTomato*^ and *Pdcd1*^*−/−*^*;Bcl11b*^*tdTomato/*+^ mice were used for scRNA-seq experiments that were done in parallel at Sanger Institute. These mice were 6–12 weeks of age at the time of experiments and were maintained at RSF of the Sanger Institute.

*Nr4a1*^*eGFPCre/*+^ mice (016617) and *Il5*^*tdTomato/*+^ (030926) mice were purchased from Jackson Laboratory. *Pdcd1*^*−/−*^*;Nr4a1*^*eGFPCre/*+^, *Pdcd1*^*−/−*^*;Il5*^*tdTomato/*+^ mice were generated by crossing *Pdcd1*^*−/−*^ mice to *Nr4a1*^*eGFPCre/*+^ mice or *Il5*^*tdTomato/tdTomato*^ mice respectively. *Nr4a1*^*eGFPCre/*+^ mice were crossed with *Il5*^*tdTomato/tdTomato*^ to generate *Nr4a1*^*eGFPCre/*+^*;Il5*^*tdTomato/*+^ mice. All mice were 6–12 weeks of age at the time of experiments and at least 3 mice of one genotype were used in each experiment. These mice were housed in the animal facility of Tongji University.

### Reagents

Fluorochrome-labeled monoclonal antibodies (clones denoted in parentheses) against B220 (RA3-6B2), CD19 (6D5), CD3 (145-2C11), CD8 (53–6.7), TCRβ (B20.6), CD49b (DX5), TCRγδ (GL3), NK1.1 (PK136), Nkp46 (29A1.4), CD11b (M1/70), CD11c (N418), Gr1 (RB6-8C5), Ter119 (TER-119), IL-7Rα (SB/199), CD25 (PC61), CD45 (30-F11), IL-33R (RWST2-2), PD-1(RMP1-30), CD69(H1.2F3) were purchased from BD biosciences, BioLegend, or eBioscience.

### Papain administration

The mice were anesthetized with 3% isoflurane and then were intranasally administered with papain (40 μg in 40 μl PBS) every day for 4 days. After the last challenge, lungs were collected and sorted or analyzed on day 0, day 7, and day14.

### Preparation of cell suspensions

Cells from the lung were prepared according to the instructions of the Lung Dissociation kit (Miltenyi Biotec, 130–095-927). The tissues were digested in a shaking water bath at 37 °C for 12 min.

### Flow cytometry assay and cell sorting

Red blood cells were removed using ACK Lysing Buffer (Gibico, A1049201). Cells were suspended in a solution of 1% (vol/vol) FBS in PBS. Before antibody labeling, Fc receptors were blocked with anti-CD16/32 (BD bioscience, 553,142). Cells were stained with antibodies on ice for 15–20 min before washing. Cells were analyzed on the Fortessa X20 cell analyzer (BD bioscience) or sorted on the FACS Aria Fusion cell sorter (BD bioscience) according to the manufacturers’ standard operating procedures. Data were analyzed with FlowJo software, version 10.

### Generation of single-cell RNA-seq library

FACS sorted ILC2s from *Pdcd1*^*−/−*^* Bcl11b*^*tdTomato/*+^ and *Bcl11b*^*tdTomato/tdTomato*^ mouse lungs were subjected to single-cell RNA-seq library preparation using smart-seq2 method [46, 73]. Briefly, single ILC2s were sorted into 96-well plates pre-filled with lysis buffer and external RNA spike-ins (Ambion) (1:500,000). First-strand synthesis and template-switching were then performed, followed by 25-cycle of pre-amplification. Complementary DNAs were purified by AMPure XP magnetic beads (Agencourt) using an automated robotic workstation (Zephyr). Quality of cDNAs was checked with the Bioanalyzer (Agilent) using high sensitivity DNA chip. Multiplex (96-plex) libraries were constructed and amplified using Nextera XT library preparation kit (Illumina). The libraries were then pooled and purified with AMPure XP magnetic beads. The quality of the library is then assessed by the Bioanalyzer (Agilent) before submission to the DNA sequencing. Pair-ended 75-bp reads were generated by HiSeq2500 sequencers.

### Read alignment and gene quantification

The pre-processed BAM files with the same indexes were first merged and converted to raw FASTQ files. The FASTQ sequences were then realigned to a modified GRCm38 mouse genome. The alignment was performed using STAR [74] and sorted by SAMtools [75] for expression quantification by featureCounts [76].

### Cell clustering and visualization

ScRNA-seq analysis was performed using Scanpy (version 1.8.1) [77] package in Python (version 3.8.8). The data were normalized and scaled to remove unwanted sources of variation according to Scanpy criteria. The normalized data was reduced the dimensionality by running principal component analysis (PCA) in Scanpy, and the first 10 principal components were used for further analysis. Computing neighbors and running Louvain clustering to identify the cell clusters and visualize them. A range of louvain resolutions was tested, and a resolution of 0.5 was used. Scanpy’s Finding marker genes function was used to find differentially expressed genes among subsets. Wilcoxon Ran sum tests were used to determine the significance of the differential expression. The gene expression and cell distribution are visualized using functions from the scanpy package.

### Using SingleR to annotate single-cell RNA-seq data

To make sure the accuracy of our data by comparing it with other activated ILC2 datasets. We used Seurat to integrate three activated ILC2 datasets from published papers (GSE122762 [48]; GSE123994 [49]; GSE117567 [50]) [38, 51] and then used integrated data as pre-labeled single-cell reference to annotate our unlabelled dataset by SingleR(1.2.4)(https://www.bioconductor.org/packages/release/bioc/vignettes/SingleR/inst/doc/SingleR.html) in R.

### Score for cell characteristics

We used the AUCell package in R to measure the level of activity of a particular collection of genes in each cell of the single-cell transcriptome in order to score each cell's characteristic activity. We begin by importing our processed cluster-labeled data into R. The gene expression ranking in each cell is determined using an expression matrix and the AUCell_buildRankings function with preset settings. Second, based on previously published research, we categorized the gene sets associated with ILC2 activation, wound healing, tissue residence, memory, proliferation, Trm, and Tcm/Tem, and then we scored each cell with various traits. Area under curve (AUC) values were computed for each gene set and cell using the AUCell_calcAUC function based on gene expression ranking. Finally, the ggplot function was used to visualize the score.

### Trajectory analysis with Monocle 3

The single-cell trajectory was constructed using the Monocle3 (version 1.2.4) package in R. To determine the effect of PD-1 KO on ILC2 activation trajectory, we used monocle3 to analyze wild-type samples and PD-1 KO samples respectively. First used the preprocess_cds function to process the data, since the processed data were imported, the norm_method parameter was set to "none". And then dimensionality reduction and cluster cells were performed. The function learn_graph is the fourth step in the trajectory building process after preprocess_cds, reduce_dimension, and cluster_cells. After learn_graph, order_cells were called. Pseudotime analysis requires the selection of a cell as an origin for the pseudotime trajectory. Origin assignment was based on the Spearman’s rank assignments for each cell. The following cells were used as origins for their respective cell-type trajectories: C1 and Cm1 ILC2s. Then the orderCells() function was used to assign cells a pseudotime value. Finally, we visualized the findings by plot_cells function.

### Gene set enrichment analysis

To identify gene signature enrichment between different samples, we used cluster profile (version 3.18.1) package in R (version4.0.2). All pathway enrichment analyses were performed using the clusterprofile (version 3.18.1) package in R (version4.0.2) to identify gene signature enrichment between different samples, and the enriched pathways were visualized.

### SCENIC analysis and visualization graphics

SCENIC [78] analysis on the scRNA-seq data without separating individual samples was performed, following the workflow using the default parameters in SCENIC R setup. The co-expression network was generated using GENIE3, and potential direct-binding targets (regulons) were based on DNA-motif analysis. And using AUCell identifies and scores gene regulatory networks or regulons in single cell level. The motif bindings were inferred based on publicly available motif binding databases provided by the S. Aerts lab. Following the regulon calculation, the individual cells’ regulon AUC values from 3.4_regulonAUC. The AUC distributions were compared and analyzed for changes in regulon activity between WT cells and PD-1 KO cells. The gene regulatory network map was created by software Cytoscape (version 3.9.1) [79].

### Statistical analysis

Data were presented as means ± SEM and were analyzed using GraphPad Prism 9 (GraphPad Software). Differences between means were analyzed by one-way ANOVA, Wilcoxon rank sum test, and unpaired T-test were used to determine the statistical significance. In the animal model study samples from individual mice were processed and analyzed separately. The representative data shown in this study represents the result from at least three independent experiments with similar results. *, *P* ≤ 0.05; **, *P* ≤ 0.01; ***, *P* ≤ 0.001; and ****, *P* ≤ 0.0001 were considered significant.

### Supplementary Information

**Additional file 1: Fig. S1.** Quality control of scRNA-seq data and the enrichment of pathways in C2 subsets.  a. FACS sorting strategies of ILC2s for scRNA-seq analysis (samples from 2 mice at each timepoint of WT or PD-1 KO mice were mixed). b-c. The violin plot showing the gene numbers, count numbers, mitochondrial ratios, and ERCC ratios of WT and PD-1 KO samples. The ERCC ratio cut-off is 25%. The mitochondrial ratio cut-off is 10%. Cells with sequencing depths greater than 2, 000 and number of genes greater than 1, 000 were used for further analysis. d. *t-SNE* plots showing T cell maker genes expressions. e-f. The bar chart shows the GO (e) and KEGG (f) pathways analysis of DEGs (*P* value <0.05 and log2FC >1) in C2 compared with C1.

**Additional file 2: Table S1.** The names of 20, 98, 257, and 243 signature genes in C1, C2, C3, and C4 respectively, which matched Fig. 1h.

**Additional file 3: Table S2.** All the signature gene sets for activation scoring (Fig. 2b), wound healing scoring (Fig. 2f), proliferation scoring (Fig. 2h), memory scoring (Fig. 2i), tissue-resident scoring (Fig. 3b), Trm scoring (Fig. 3d), Tcm/Tem scoring (Fig. 3e).

**Additional file 4: Fig. S2.** The transcriptomic signature of C3 cells. a. The top 20 DEGs in C4 and C3 cells respectively. b-c. The bar chart shows the GO (b) and KEGG (c) pathways analysis of DEGs (*P* value <0.05 and log2FC >1) in C3 compared with C4.

**Additional file 5: Fig. S3.** The PD-1 deletion effects on ILC2. a. t-SNE plot showing the distributions of WT cells in the re-clustered integrated data (341 WT and 362 KO). b. Stacked violin plots showing the distributions of expression of ILC2-associated genes. The y-axis indicates the log2 (normalized count + 1) expression levels. c. The bar chart shows the KEGG pathways analysis of DEGs (*P* value <0.05 and log2FC >1) in Cm4 of PD-1 KO compared with WT. d. GSEA analysis of DEGs between PD-1 KO versus WT Nr4a1^+^ILC2s (*p*<0.05). e. The bar chart shows the GO (left) and KEGG (right) pathways analysis of DEGs (*P* value <0.05 and log2FC >1) in the non-activation cells between PD-1 KO and WT. f. The bar chart shows the GO (left) and KEGG (right) pathways analysis of DEGs (*P* value <0.05 and log2FC >1) in the Cm2 between PD-1 KO and WT. g. Histogram showing the composition of the clusters within different time points samples.

## Data Availability

The raw scRNA-seq data of this study have been deposited in the European Nucleotide Archive database and are accessible through the accession number PRJEB65049. The trimed sequencing metrics of scRNA-seq data has been deposited on https://github.com/Yuzhang01010101/ILC2.All other relevant data are available from the corresponding author on request.

## Abbreviations

ILC2s: Group 2 innate lymphoid cells
Trm: Tissue-resident memory T cell
Tem/Tcm: Effector/central memory T cell
PD-1: Programmed cell death protein-1
scRNA-seq: Single-cell RNA-sequencing
t-SNE: T-Distributed Stochastic Neighbor Embedding
DEGs: Differentially expressed genes
Log2FC: Log2 fold change
