# Supplementary figures and images for: Nr4a1 marks a distinctive ILC2 activation subset in the mouse inflammatory lung
Source: BMC Biol. 2023 Oct 13;21:218. doi: 10.1186/s12915-023-01690-3 (PMC10576290; doi:10.1186/s12915-023-01690-3)

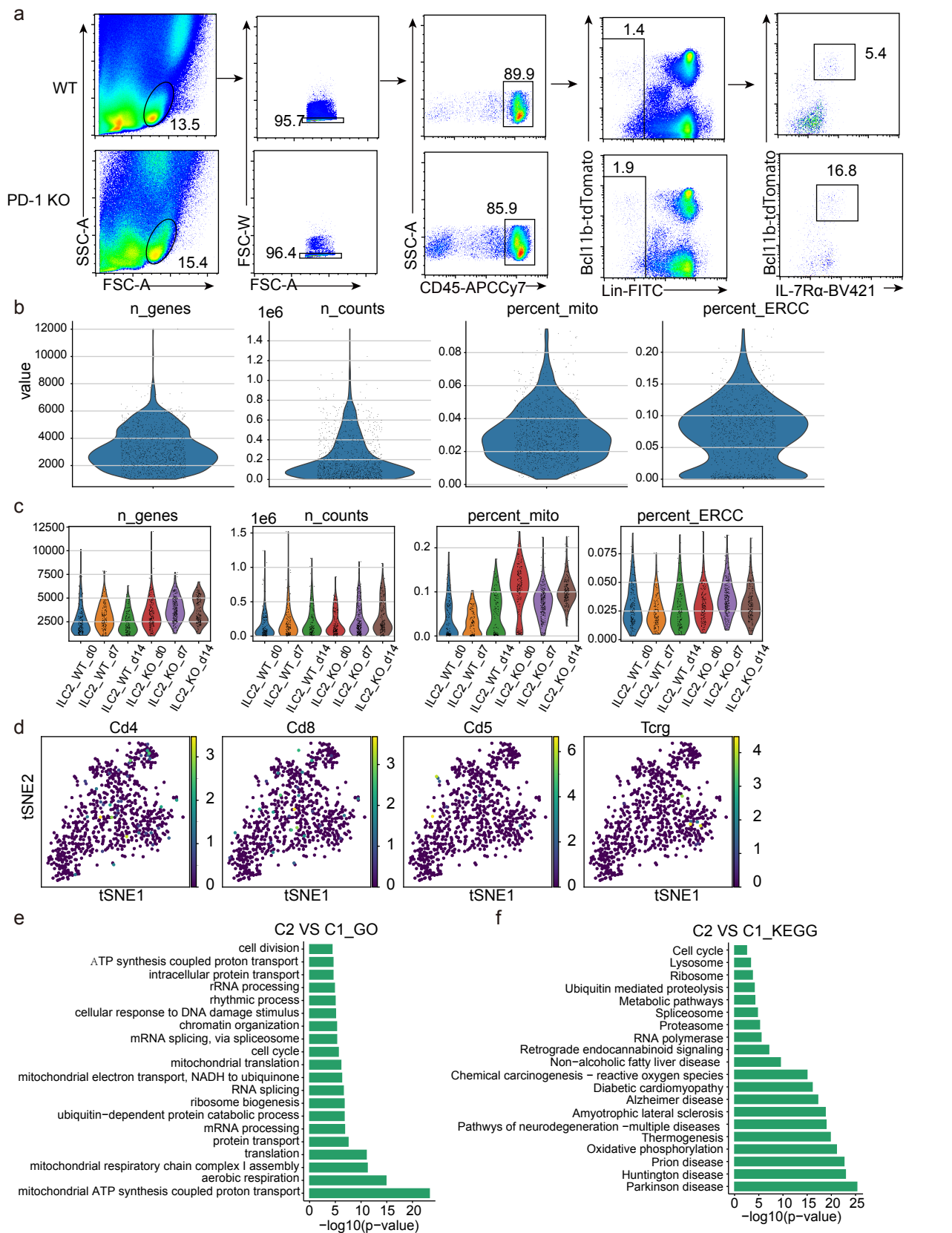

Supplement: Supplementary file 1 — Additional file 1: Fig. S1. Quality control of scRNA-seq data and the enrichment of pathways in C2 subsets. a. FACS sorting strategies of ILC2s for scRNA-seq analysis (samples from 2 mice at each timepoint of WT or PD-1 KO mice were mixed). b-c. The violin plot showing the gene numbers, count numbers, mitochondrial ratios, and ERCC ratios of WT and PD-1 KO samples. The ERCC ratio cut-off is 25%. The mitochondrial ratio cut-off is 10%. Cells with sequencing depths greater than 2, 000 and number of genes greater than 1, 000 were used for further analysis. d. t-SNE plots showing T cell maker genes expressions. e-f. The bar chart shows the GO (e) and KEGG (f) pathways analysis of DEGs (P value <0.05 and log2FC >1) in C2 compared with C1. [file 12915_2023_1690_MOESM1_ESM.pdf]

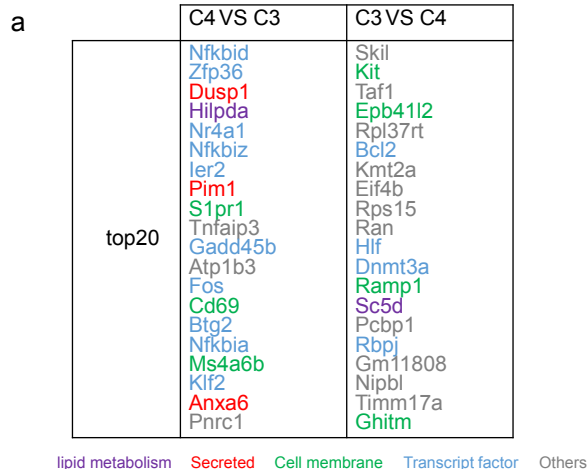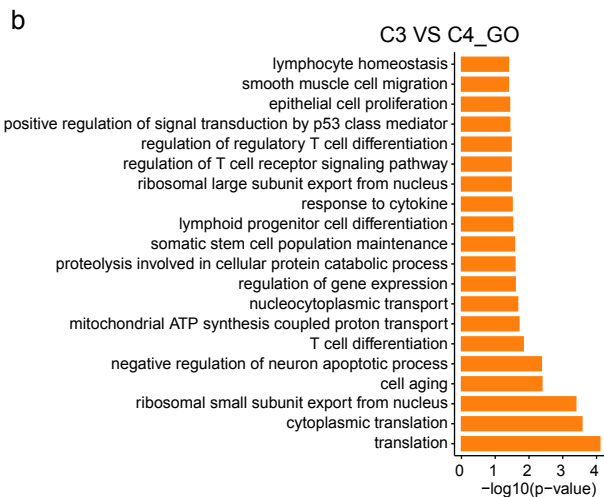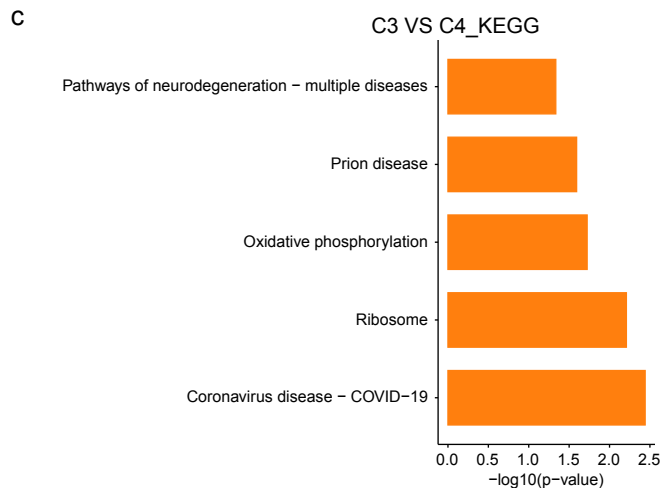

Figure S2

Supplement: Supplementary file 4 — Additional file 4: Fig. S2. The transcriptomic signature of C3 cells. a. The top 20 DEGs in C4 and C3 cells respectively. b-c. The bar chart shows the GO (b) and KEGG (c) pathways analysis of DEGs (P value <0.05 and log2FC >1) in C3 compared with C4. [file 12915_2023_1690_MOESM4_ESM.pdf]

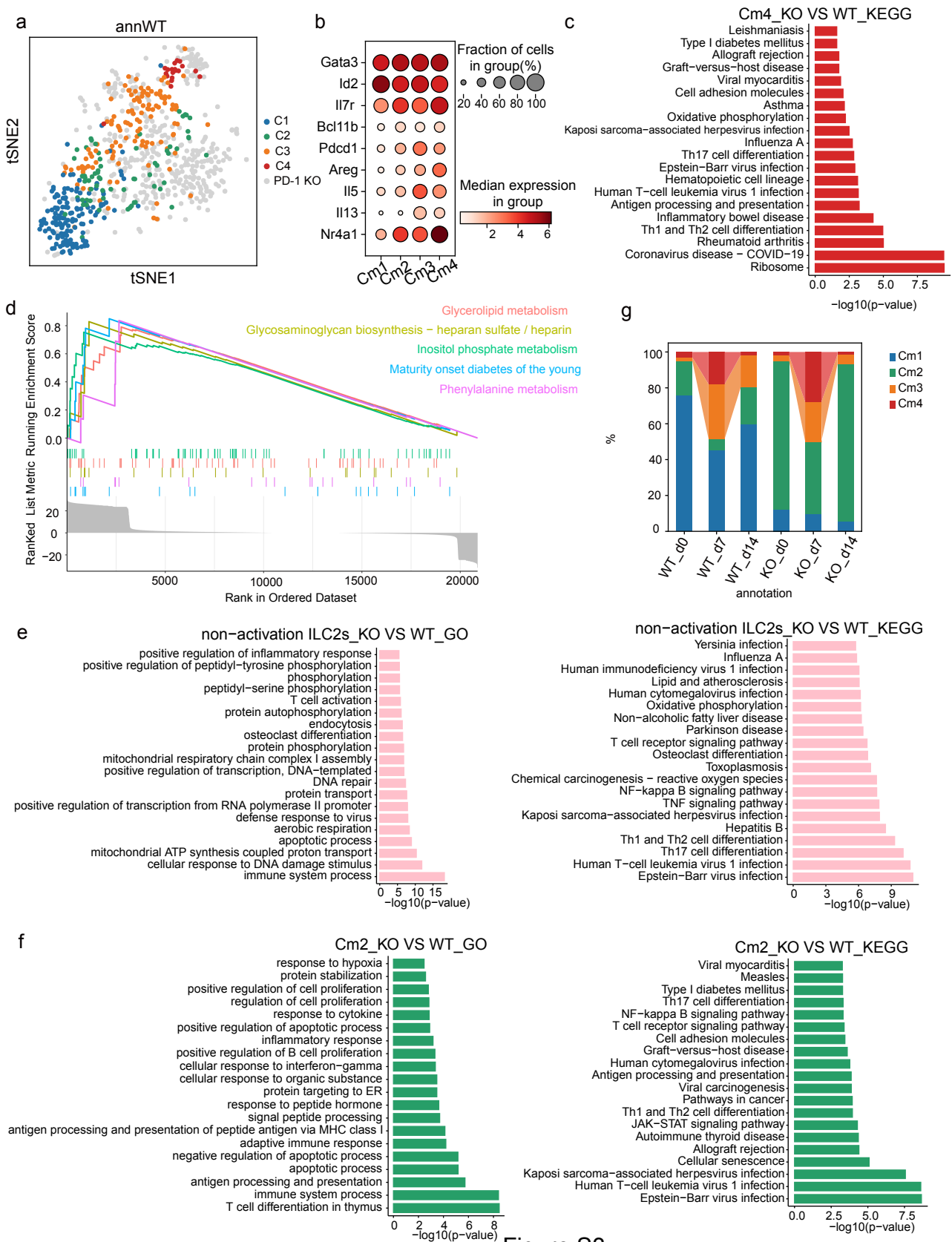

Figure S3

Supplement: Supplementary file 5 — Additional file 5: Fig. S3. The PD-1 deletion effects on ILC2. a. t-SNE plot showing the distributions of WT cells in the re-clustered integrated data (341 WT and 362 KO). b. Stacked violin plots showing the distributions of expression of ILC2-associated genes. The y-axis indicates the log2 (normalized count + 1) expression levels. c. The bar chart shows the KEGG pathways analysis of DEGs (P value <0.05 and log2FC >1) in Cm4 of PD-1 KO compared with WT. d. GSEA analysis of DEGs between PD-1 KO versus WT Nr4a1+ILC2s (p<0.05). e. The bar chart shows the GO (left) and KEGG (right) pathways analysis of DEGs (P value <0.05 and log2FC >1) in the non-activation cells between PD-1 KO and WT. f. The bar chart shows the GO (left) and KEGG (right) pathways analysis of DEGs (P value <0.05 and log2FC >1) in the Cm2 between PD-1 KO and WT. g. Histogram showing the composition of the clusters within different time points samples. [file 12915_2023_1690_MOESM5_ESM.pdf]
